# Visualizing Composition and Functionality of Porous Catalysts Using Dual-Emissive Fluorescent Nanoprobes

**DOI:** 10.1021/acscentsci.4c02039

**Published:** 2025-03-12

**Authors:** J. J. Erik Maris, Yadolah Ganjkhanlou, Caroline Versluis, Rafael Mayorga González, Nikolaos Nikolopoulos, Freddy T. Rabouw, Eelco T. C. Vogt, Bert M. Weckhuysen, Florian Meirer

**Affiliations:** † Inorganic Chemistry and Catalysis, Institute for Sustainable and Circular Chemistry, 8125 Utrecht University, Universiteitsweg 99, 3584 CG Utrecht, The Netherlands; ‡ Soft Condensed Matter, Debye Institute for Nanomaterials Science, Utrecht University, Princetonplein 1, 3584 CC Utrecht, The Netherlands

## Abstract

The performance of heterogeneous catalyst bodies is closely related to the accessibility and properties of their catalytically most active regions. Visualizing and understanding the composition and functionality of these multicomponent hierarchically structured materials is often time-consuming and expensive. To overcome these challenges, fluorescent nanoprobes have been employed to visualize heterogeneous, complex material composition. So far, fluorescence microscopy approaches are limited to the measurement of spatial heterogeneities of only one property—such as accessibility or acidity—in a single measurement. In this work, we introduce a dual-emissive solution containing carbon dots and porphyrin nanoprobes to simultaneously map different material domains and properties within heterogeneous catalyst particles. We investigated catalyst components (oxides and supports), spray-dried cracking catalysts, and extruded catalysts. The selective adsorption as well as the aggregation of the probes affect their fluorescence emission characteristics, allowing us to identify regions with similar properties and composition. Our multiprobe staining method is a facile approach for high-throughput composition and property mapping of porous materials, including solid catalysts and adsorbents.

## Graphical abstract

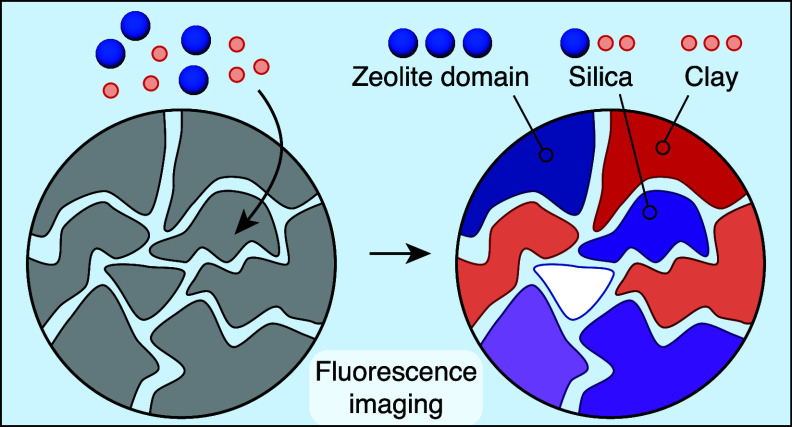

## Introduction

Catalysis is a pillar of modern society as it is essential for the production of everyday products, such as fuels, pharmaceuticals, food ingredients, and plastics.^1,2^ However, catalysis faces new challenges with the transition from fossil-feedstock-based to more sustainable, circular production processes.^3,4^ Therefore, testing and development of novel heterogeneous catalysts that can enable these new processes is more important than ever.^1,5^ Solid catalysts are often porous and have heterogeneous composition and, therefore, consist of domains with different properties, such as acidity, wettability, and pore-network accessibility. For the development of new catalysts or optimization of existing ones, rapid, facile, and high-throughput characterization of these functional domains is required. Here, a short feedback loop between the catalyst functionality/composition and synthesis parameters has the potential to boost catalyst development. Standard characterization techniques require intricate sample preparation and/or advanced analytical techniques, such as electron or X-ray imaging, making them often costly, complicated, and time-consuming.^6–9^

An alternative and highly complementary approach to map functional porous materials is by staining their accessible pore space with fluorescent nanoprobes. These reporters diffuse into the pores and interact with and adsorb to the pore walls. After equilibrium has set, the probes are imaged with confocal laser scanning microscopy (CLSM) in a field of view over 100 μm × 100 μm with submicrometer resolution. This technique allows a reconstruction of the catalysts’ functional domains by stacking two-dimensional slices into a three-dimensional volume. CLSM—and microspectroscopy, in a broader sense—have been important for the identification and characterization of catalytically active domains,^10–16^ as well as mass transport through porous catalyst bodies.^17–23^ However, the outlined approach is not limited to porous catalysts and has also been applied for biological samples^24,25^ and mineralogical samples for geology.^26,27^

Currently, staining approaches map only one property of porous solids (e.g., accessible porosity) based on changes in a specific region of the fluorescence emission spectrum (e.g., overall intensity). However, more information can be extracted when a set of fluorescent probes is used with complementary interactions and/or adsorption properties. Particularly, zeolite catalytic activity and pore-network accessibility of individual ∼100-μm-sized fluid-catalytic-cracking (FCC) particles have been mapped by performing two subsequent staining steps with different fluorogenic and fluorescent molecules.^11,13^ Even though this multimodal approach visualizes the zeolite domains and FCC matrix, it is limited by the requirement for two different staining experiments (sometimes even on different particles) and does not discriminate between the different constituents of the FCC matrix.

We build on these earlier studies and introduce a set of fluorescent reporters that can identify various material domains within a catalyst particle in only a single measurement. The probe mixture can be synthesized easily from an inexpensive and nontoxic glutathione precursor in a one-pot solvothermal synthesis, followed by purification via dialysis.^28–30^ In an earlier study, we showed that the fluorescent nanoprobes in this system are a separable mixture of 4-nm carbon-dot (CD) nanoparticles and 1–2 nm water-soluble porphyrin (PP) derivatives (see Figure 1a, as well as Figures S1a and S1b).^30^ The system is easy to use, as both fluorescent species can be excited directly at 405 nm, making calibration of the multiple excitation intensities unnecessary. The CDs and PP emit in two different wavelength regimes in which we define three bands (Figure 1b). These emission properties allow for the extraction of sensory information via the intensity of the emission bands, independently of the probe concentration, as is depicted in Figures 1c and 1d.

**Figure 1:**
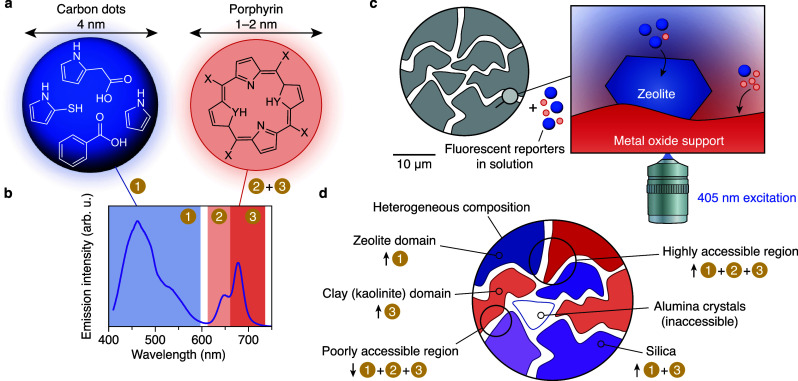
Smart probes for mapping heterogeneous catalysts. (a) The fluorescent reporters investigated in this work are carbon-dot (CD) nanoparticles and water-soluble porphyrin (PP) molecules. Here, X represents polar functional groups such as COOH, OH, SH, phenol, and glutamate, whereas YH represents S or NH. The CD fluorophores are example structures, and others can be formed during synthesis as well. (b) The fluorescence emission spectrum of the CD/PP mixture in aqueous solution contains separate emission bands when exited at 405 nm. The numerals 1–3 indicate the spectral features. (c) A porous solid with an unknown composition can be studied via the sorption of fluorescent reporters to the pore walls. Preferential sorption and/or response of the fluorescence emission spectrum to specific material properties provides contrast to discriminate, e.g., a zeolite domain and the metal-oxide support material. (d) Heterogeneous material properties including wettability are probed by the relative strength of one or more CD/PP fluorescence emission bands (arrows up). Additionally, the solid’s accessible porosity for the probes relates to the absolute strength of the emission bands.

## Results and Discussion

The fluorescent response of the dual-emissive CD–PP mixture is different for materials with different (surface) properties, such as points of zero charge and wettability. For most catalyst components (metal oxides and support materials), the response is unique over the full visible wavelength range. In Figure 2a, their fluorescence emission spectra are given, which were recorded on the surface or inside the host material using CLSM. These spectra show that many of the metal oxides have distinct spectral features allowing them to be identified based on the fluorescence response of the adsorbed CDs and PP. We demonstrate this feature in Figure 2b with a true-color image of a heterogeneous FCC catalyst particle. Such cracking catalysts have recently been shown to be promising for large-scale applications in sustainable processes, such as biomass cracking and plastic recycling.^31–35^ Laboratory-made FCC particles were prepared with and without kaolinite clay using the same spray-drying method. The stained clay is red-emitting (Band 3 in Figure 2a), whereas stained ultrastable Y (USY) zeolite emits in the blue (Band 1) and stained silica emits both in the blue and red (Bands 1 + 2 + 3). The alumina domains in this catalyst particle are nonporous and appear in black—similar to the pores inside the particle, which cannot be discriminated in the true-color image (Figure 2b). A clear difference in material distribution is observed for the two samples, and the zeolite domains can be well-resolved. Thus, already without data analysis, the spectral differences allow for direct identification and interpretation of the spatial distribution of the zeolite, metal oxide, and clay material domains (even in three dimensions; see Figure S2).

We obtain a deeper understanding of the sensory information encoded in the fluorescence emission of the CD–PP mixture by comparing its emission spectra for various metal oxides and kaolinite clay host materials. The wettability, or polarity, of the host material leads to a different uptake or surface coverage of the CD and PP. This is a result of the different polarity of the two fluorescent probes. The affinity of the CDs and PP for hydrophobic and hydrophilic liquids, respectively, is demonstrated in Figures S1a and S1b. The same affinity was found for staining a solid host material. Two stained porous silica particles with different surface properties are shown in Figure 2c. Without calcination pretreatment, the silica particle is hydrophilic due to the OH terminated silica; however, heating leads to bridging of the OH groups on the silica surface, making it more hydrophobic.^36,37^ The different surface properties are readily visible in the true-color image, where the signal of adsorbed CDs on the hydrophobic host increases dramatically. In contrast, PP is known to have a high affinity for hydrophilic clays.^38,39^ Thus, hydrophobic/amphiphilic host materials lead to a relatively high CD signal, and vice versa, hydrophilic host materials result in a relatively low CD signal (Figure S3).

**Figure 2:**
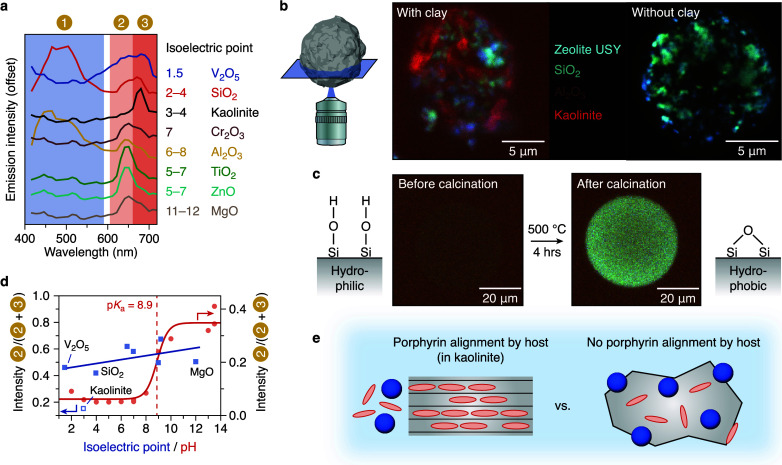
Fluorescence of the carbon-dot/porphyrin mixture for metal-oxide host identification. (a) Fluorescence emission spectra recorded with confocal laser scanning microscopy (CLSM) at the surface or interior pore network of different metal oxides with different isoelectric points (Table S4). Images were recorded after staining by a carbon-dot (CD) and porphyrin (PP) mixture (0.5 mg/mL in 25 mM PBS buffered at pH 7; λ_ex_ = 405 nm). (b) True-color CLSM image of two laboratory-made FCC particles prepared via spray drying after staining with the CD–PP mixture imaged at ∼5 μm into the sample. The FCC particles are composed of silica, ultrastable Y (USY) zeolites, and alumina, and either contain (left), or do not contain kaolinite clay (right). (c) True-color CLSM image of a spherical silica particle stained before (left) and after calcination at 500 °C (right). The observed change in fluorescence emission is due to a change in surface wettability, which alters adsorption affinity of the CDs to the silica surface. Both images were recorded with the same settings. (d) Intensity ratio of PP bands 2/(2 + 3) as a function of the solution pH (circles, raw data in Figure S6) and isoelectric point of metal oxides (squares). The straight line is a fit of the isoelectric point of the metal oxides (solid squares) and the intensity band ratio, whereas the red line is a fit of the dissociation equilibrium of a weak acid as a function of the pH. (e) Schematic representation of PP alignment in the layered structure of kaolinite clay, leading to J-aggregation.

In addition to the wettability of host solid, its fluorescence response after staining is dependent on aggregation of the PP. It has been reported before that PP is pH-responsive in aqueous solution.^40^ Moving from acidic and neutral to basic conditions, the emission maximum shifts toward the blue, causing a sudden jump in the 2/(2 + 3) band ratio (red data points in Figure 2d and Figure S1c). Here, a change in molecular properties of the PP (e.g., charge, H-bond-formation capacity, etc.) leads to molecular aggregation at the p*K*_a_ (see Figures S4 and S5). Protonation of the PP’s functional groups and PP aggregation could both cause the observed red shift, because the two happen simultaneously around the p*K*_a_. However, the 2/(2 + 3) band ratio is barely dependent on the isoelectric point of the host material, which is a measure of the pH around the solid (blue data points in Figure 2d). This is a strong indication that aggregation on the host’s surface rather than the local pH around the host causes the red shift in the fluorescence emission.

A special case is the CD/PP fluorescence emission in kaolinite clay, which provides further insight into the PP fluorescence behavior. The stained kaolinite has bright fluorescence emission around 680 nm (Band 3, Figure 2a), which is reflected in a low 2/(2 + 3) intensity band ratio (Figure 2d). The layered structure of kaolinite allows the planar porphyrin molecule to enter, while the carbon dots are size-excluded (Figure 2e).^41,42^ As a result, kaolinite clay has no CD fluorescence emission (Band 1). Because of geometrical constraints, the PP aligns head-to-tail in the pores of kaolinite, leading to aggregation, as is depicted in Figure 2e. This type of aggregation (J-aggregation) is characterized by a red shift in fluorescence emission.^43,44^ The aggregation results in the same spectral shift as we have seen previously in aqueous solution (Figure S4). This corroborates that the red shift in PP fluorescence is caused by J-aggregation. In addition, the formation of large aggregates that quench the fluorescence is suppressed inside the kaolinite, boosting the measured signal relative to free solution where aggregation can occur (Figure S4b and ref 21). This makes the CD–PP system particularly sensitive to layered, porous (clay) materials.

The contrast of our dual-emissive nanoprobes is based on the wettability, surface properties, and pore structure of the host material. All these factors determine the adsorption and aggregation of the CD and PP as well as the probes’ ability to travel into the material domains. A complete overview of the advantages, limitations, and applications of dual-emissive probes is given in Table S5.

Properties, such as material domain size, shape, and distribution, can be extracted from the CLSM hyperspectral images on the single-particle level. We use principal component analysis followed by *k*-means clustering to generate a material map (see comments to Figures S7, S8). The sample presented here is a laboratory-made extruded catalyst body containing the same components as the FCC particle shown before. These extrudates are millimeters to centimeters in size and can be imaged at different length scales with CLSM (see e.g., ref 20). The reconstructed material map in Figure 3b matches well with the true-color CLSM image in Figure 3a. Interestingly, even “overlap” domains with both zeolite and kaolinite, where the measured spectra are a mixture of these individual components, can be identified. We stress that such a map—with a large field of view—cannot simply be acquired with other techniques, e.g., energy dispersive X-ray spectroscopy/scanning electron microscopy (EDX/SEM). In the case of EDX/SEM, small changes in the elemental composition and crystal structure have little contrast and are extremely challenging to identify.

The material map allows for further quantitative analysis by filtering out the zeolite domains (Figure 3c) from which their size distribution is computed (see Figure 3d, as well as Figure S9). In SEM images of the pristine zeolite (Figure S10), we observe micrometer-sized agglomerates of intergrown zeolites comprised of submicrometer-sized crystallites.^45^ As shown in Figure 3d, the individual crystallites are in 0.2–1.2 μm in size. Thus, the presence of zeolite domains >1.2 μm in the extrudate—measured with CLSM—shows that the agglomerates in the pristine zeolite did not fully break up into the individual crystallites during the extrusion process. Note that our laboratory extrusion process is extremely low shear compared to industrial extrusion.

## Conclusions

Altogether, we have introduced a set of fluorescent CDs and fluorophores that enable the characterization of metal-oxide and clay components commonly found in heterogeneous catalysts and mineralogical samples. It not only maps accessibility but also provides information about local properties of the studied material, such as surface wettability and material-domain shape. This information is complementary to high-resolution X-ray and electron-microscopy techniques,^6–9^ which typically visualize catalyst particle cross sections, or at best, particle volumes rather than the pore surface the reactant interacts with. The reported sensing capabilities could be useful when optimizing solid catalyst and adsorbent technologies, e.g., to identify hydrophobic adsorbents for CO_2_ capture that are resistant to water poisoning.^46^ Moreover, we see potential to characterize other supported catalysts, e.g., to monitor the metal-nanoparticle loading throughout a catalyst particle. The facile probe synthesis and aqueous-solution-based staining procedure, in combination with off-the-shelf CLSM, make our method easy to implement for both academic and industrial research groups.

**Figure 3:**
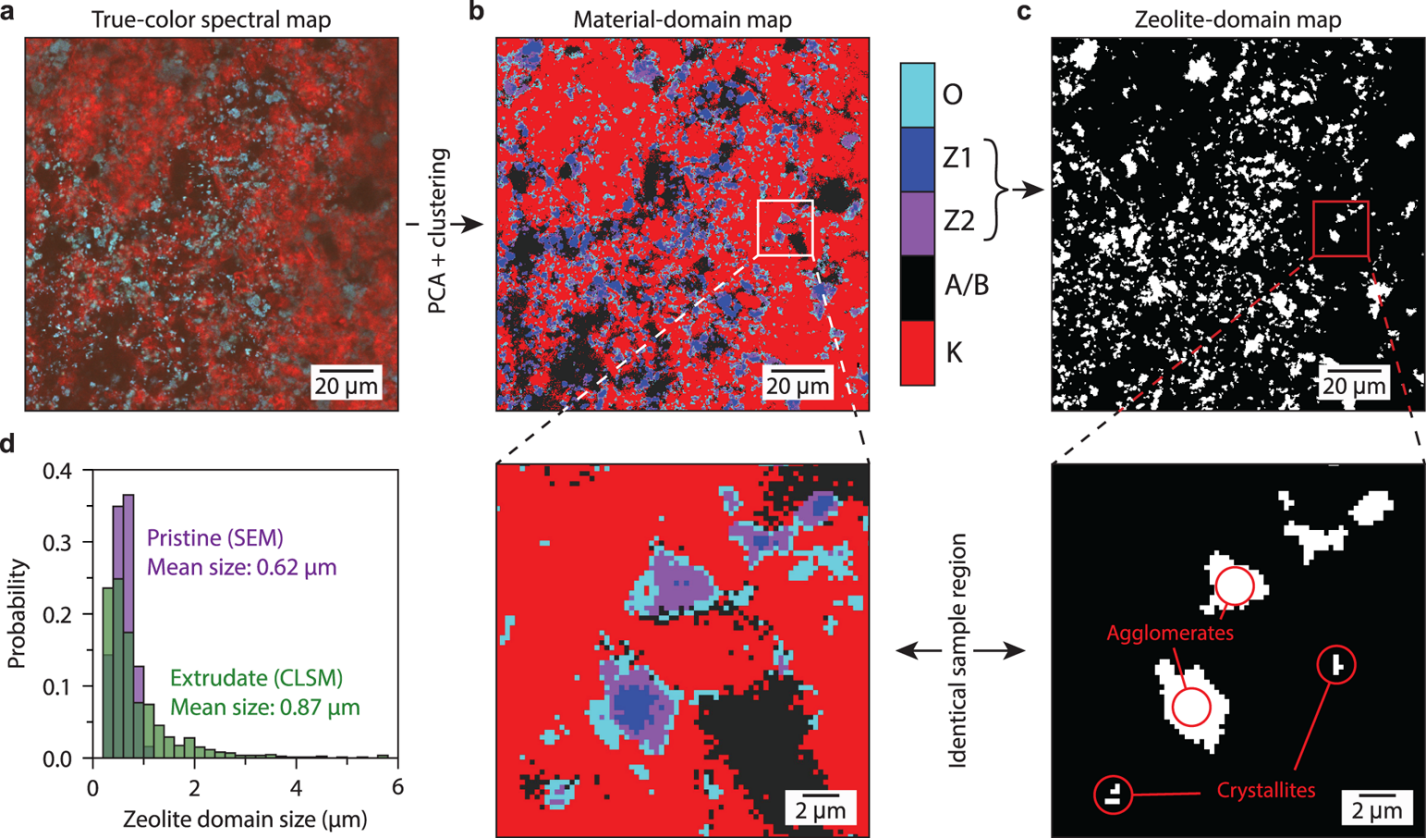
Host material identification in the extrudate samples. (a) True-color confocal laser scanning microscopy (CLSM) image of an extrudate (Ex.1 in Table S1)—containing USY, clay, and alumina binder—after staining by a carbon-dot–porphyrin solution (buffered at pH 7; λ_ex_ = 405 nm). (b) Material map obtained via principal component analysis (PCA) followed by *k*-means clustering of the data in panel (a). A magnification is shown in the inset below. The spectra were assigned to kaolinite (K), alumina and/or background pores (A/B), zeolite (Z1 and Z2), and overlap of zeolite and kaolinite (O). (c) Map of zeolite domains constructed from zeolite clusters in panel (b). Only zeolite domains with a size >2 pixels are shown. Holes in the domains were filled artificially. A magnification of the zeolite map is given in the inset. Sharp facets can be observed for some isolated zeolite domains. (d) Histogram of zeolite domain size of map in panel (c) overlaid with sizes of the pristine, submicrometer-sized zeolite crystallites obtained with scanning electron microscopy (SEM).

## Supplementary Material



## Notes

Additional experimental details, materials, and methods as well as supplementary figures and tables (PDF)
