# Supplementary material for: Visualizing Composition and Functionality of Porous Catalysts Using Dual-Emissive Fluorescent Nanoprobes
Source: ACS Cent Sci. 2025 Mar 12;11(6):872–7. doi: 10.1021/acscentsci.4c02039 (PMC12203431; doi:10.1021/acscentsci.4c02039)
Supplement: Supplementary file 1 [file oc4c02039_si_001.pdf]

Supplementary information

Visualizing Composition and Functionality of Porous Catalysts using Dual-Emissive Fluorescent Nanoprobes

J.J. Erik Maris,[1,+] Yadolah Ganjkhanlou,[1,+] Caroline Versluis,[1] Rafael Mayorga González,[1] Nikolaos Nikolopoulos,[1] Freddy T. Rabouw,[1,2] Eelco T.C. Vogt,[1] Bert M. Weckhuysen,[1] Florian Meirer[1,*]

[+] These authors contributed equally to this work.

[1] Inorganic Chemistry and Catalysis, Institute for Sustainable and Circular Chemistry, Utrecht University, Universiteitsweg 99, 3584 CG Utrecht, The Netherlands

[2] Soft Condensed Matter, Debye Institute for Nanomaterials Science, Utrecht University, Princetonplein 1, 3584 CC Utrecht, The Netherlands

**Corresponding author:* f.meirer@uu.nl

1 Contents

2 Chemicals, Materials and Methods 3

3 Supporting Figures and Table 7

4 References 20

2 Chemicals, Materials and Methods

Chemicals and materials. $Al(OH)_3$ (Sigma Aldrich, reagent grade, 239186), fumed silica (Sigma Aldrich, surface area of 200 m^2/g, S5505), kaolinite (Sigma Aldrich, 03584), SiO_2 particles (AGC Si-Tech Co., Ltd, SUNSPERA), USY zeolite (Zeolyst, CBV780; surface area, porosity, and Si/Al=40). All chemicals were used as received with no further purification except water, which in all cases was purified through a Milli-Q system to a resistivity of 18.2 MΩ·cm. H_3PO_4 (Sigma Aldrich, 695017, 85%), NaH_2PO_4 (Sigma Aldrich, S0751), Na_2HPO_4 (Sigma Aldrich, S9763), NaOH (Sigma Aldrich, 655104, ≥97%) and solution used for including 2-Pentanol (Sigma Aldrich, P8017), THF (≥99.9%, 186562), anhydrous acetic acid (A6283, ≥99%) were all of analytical grade and used without any further purification.

Synthesis and dialysis of carbon dots and porphyrin mixture. The carbon-dot (CD)/porphyrin (PP) mixture (PP) synthesis was reproduced from our earlier work.[1] In short, reduced *L*-glutathione 98% (GSH Sigma Aldrich, 3541) was dissolved in formamide (FA, Sigma Aldrich 99%, 4610-OP) at room temperature. The solution was poured in a Teflon-lined autoclave (Parr 4749) and heated at 180 °C for 3 h. The obtained solution was filtered through a 0.22-µm filter and centrifuged at 5000 rpm for 5 min to remove large aggregates. The supernatant solution was dialyzed using a 1 kDa dialyzing membrane (Spectra/Por 7 Membrane Tubing, MWCO 3.5 kDa, Fisher Scientific) for 1 week, while the water was replaced daily. The progress of the separation was followed via the luminescence of the dialysate (excitation with a 5 mW, 405-nm laser pointer). Approximately 7 days of dialysis were sufficient to reduce the concentration of luminescent species in the dialysate below a level observable by eye, and the dialysis was stopped.
To harness the pH-sensitivity of the PP, it is important that the PP is in free solution and not encapsulated in the CD nanoparticles, which was achieved by keeping the solvothermal synthesis time short (**Figure S12**).

Preparation of catalyst extrudates. Extrudates containing kaolinite, $Al(OH)_3$, ultrastable Y (USY) zeolite, and silica were prepared. First, the raw materials were weighted and mechanically mixed for 5 min to obtain a homogeneous mixture. Then 1M HCl was added dropwise to the mixture to obtain a viscous gel. Usually, a 1:1 weight ratio of solution to solid is sufficient to obtain gel with desired viscosity. The gel was then transferred into 5 mL syringe and extruded through the syringe opening (without needle) on a glass coverslip. The obtained catalyst bodies were dried at 60 °C overnight and calcined at 550 °C to obtain the final extrudates. In total, four extrudates with a composition given in **Table S1** were prepared. The specific surface area and mean pore size of the raw materials can be found in **Table S2**.

Table S1 | Composition of the prepared catalyst extrudates materials.

Sample	Weight (g)			
	Kaolinite	SiO_2	$Al(OH)_3$	USY
Extrudate 1	2.0	—	0.5	0.50
Extrudate 2	0.50	—	0.5	—
Extrudate 3	—	0.50	0.50	—
Extrudate 4	0.51	—	0.33	0.33

Table S2 | Physicochemical properties of the prepared catalyst extrudate materials. The specific surface area and mean pore size were measured with nitrogen physisorption (methods in section Characterization).

Compound	Specific surface area (m^2/g)	Mean pore size (nm)
CBV-780	729	29
SiO_2	340	30
Alumina	3	—
Kaolinite	10	4.5

Preparation of spray-dried catalyst particles. A slurry was prepared, which was spray-dried to obtain the product. First, the raw materials were mixed and gradually added to 195 ml of H_2O. The solid content in the slurry was about 20 wt%. Anhydrous acetic acid was added to the slurries to reach pH = 3. The acidity is required to dissolve the alumina binder. The prepared slurries were spray-dried using a spray dryer (Buchi B-290) with an input temperature of 170 °C and output temperature of approximately 117 °C. The aspirator was set to 100 and the gas spray to 35. The composition of the slurries used to synthesize the spray-dried particles is given in **Table S3** and scanning electron microscopy images in **Figure S13**.

Table S3 | Composition of the prepared slurries to make spray-dried catalyst particles.

Sample	Fraction (wt%)			
	Kaolinite	SiO_2	Al_2O_x	USY
Slurry 1	36	6	38	20
Slurry 2	—	12.7	67.3	20

Standard solutions. The solution pH is an important factor that was optimized to maximize the resolvability of the catalysts' materials domains. We found that pH = 7 resulted in the highest contrast and resolvability in the tested materials (**Figure S14**). Moreover, pH buffering of the staining solution improved spatial resolvability, as it reduces the range at which the solid affects the pH near its surface (**Figure S15**). Therefore, unless otherwise stated, the solutions used in this work contained 0.5 mg/mL CD/PP powder in 25 mM sodium PBS aqueous solution, buffered at pH = 7. Where necessary, the pH of the buffer was adjusted using 0.5 M H_3PO_4 (acidic) or NaOH (basic) solutions. Please note the use of a sodium PBS buffer, as different aggregation behaviour of the CD/PP mixture was observed with a potassium PBS buffer.

Characterization. UV-Vis spectra were recorded using a UV-Cary 200 spectrophotometer. Photoluminescence emission spectra in Figure 1b were recorded with a Jasco spectrofluorometer (FP 8300) at 200 nm/min. The photoluminescence spectra in all other figures were recorded using AvaSpec-ULS2048CL-EVO as a spectrophotometer and AvaLight-HPLED-405 as a light source with wavelength of 405 nm. All emission spectra were recorded at 405 nm excitation wavelength unless otherwise noted. Fourier-transform infrared (FT-IR) spectra have been measured in attenuated total reflectance (ATR) mode by a PerkinElmer 2000 instrument on a droplet of CDs with a concentration of 1 g/L. X-ray diffraction (XRD) patterns have been recorded by D2 Brucker diffractometer in the 2ϑ range of 10–40° using CoKα source and a slit size of 1 mm. Nitrogen adsorption/desorption isotherms of the extrudate materials were measured at −196°C on a Micromeritics TriStar II Plus. The surface area and micropore volume were computed using BET plot and the *t*-plot methods respectively using the Micrometrics MicroActive software. The electron microscopy images were recorded with a FEI Helios NanoLab G3 UC scanning electron microscope (SEM) operated in immersion mode with an acceleration voltage of 2 keV and a current of 25 pA. The zeolite crystallite size was determined manually using ImageJ.[2] For direct comparison between particle sizes determined via SEM and (confocal) fluorescence microscopy, please note that spreading out of the recorded signal because of the lateral resolution (i.e., a few hundred nm) of fluorescence microscopy can lead to an overestimation of the particle size.

Confocal laser scanning microscopy. Confocal laser scanning microscopy (CLSM) was utilized for colorimetric analysis of stained model systems. The CLSM measurements were performed using a Nikon A1 confocal microscope configured with an Eclipse Ti2-E inverted microscope body and equipped with a 405 nm solid-state laser source and a Nikon oil immersion objective (Nikon CFI Plan Apo Lambda 60x Oil, NA = 1.4) was used with a Nikon type F immersion oil. A spectral analyser in the Nikon A1 system equipped with 32 photomultiplier tubes (PMTs) was used to collect the hyperspectral images. Here, the spectra were recorded with bins of 10 nm (415–735 nm) with a corresponding excitation wavelength of 405 nm. A typical hyperspectral image contained 1024×1024 pixels with a pixel size of 150 nm. The scan speed was 1/8 frame/s, we used 2× line integration and a pinhole of 1.5 AU (@405nm) which is equivalent to 37 µm.

Since the staining procedure does not require heating, an oil objective is used to obtain enhanced resolution and contrast.

Data analysis of the CLSM hyperspectral images. The recorded hyperspectral CLSM images were pre-processed, and the spectra were plotted in CLSM Spectra Lab v. 0.30, which is a home-built software available complementary to this work. Principal component analysis (PCA) as well as *k*-means clustering refined by Gaussian mixture modelling was done using XANES Wizard v. 3.1.0 which is part of the TXM-Wizard software suite.[3]

The pre-processing in CLSM Spectra Lab consisted of four steps: 1. background subtraction; 2. binning; 3. masking; 4. normalization; and 5. exporting data to the XANES wizard. A detailed instruction is provided with the CLSM Spectra Lab Software (*Instruction CLSM Spectra Lab for PCA + clustering in XANES Wizard; For version 0.30*).

1. A background correction was done with a hyperspectral dark reference spectrum recorded without an excitation source (laser shutter closed). This spectrum was subtracted on a per-pixel basis from the hyperspectral image to perform the background correction.
2. To reduce the noise in the single-pixel spectra, the pixels were binned into 2×2 clusters.
3. The hyperspectral image was masked into foreground and background. The first principal component (computed with principal component analysis including all dimensions) was segmented based on an automatic threshold using Otsu's method, or by setting a manual threshold. This effectively masked the hyperspectral images based on the summed intensity per pixel. In addition, saturated pixels were masked out to the background and not included in the analysis and plotting. This step was skipped for hyperspectral images that were exported to XANES Wizard for clustering analysis.
4. The spectrum in each pixel was normalized to its maximum intensity. This ensured that the clustering was performed based on spectral features and not on the absolute intensity.
5. The normalized hyperspectral images were imported in XANES Wizard and clustered after dimensionality reduction with a varying number of principal components (more information in Notes to **Figure S8**). *K*-means clustering was done, which was optionally refined with expectation maximization (EM) for a Gaussian mixture model (GMM). The non-normalized spectra of the clusters were subsequently plotted using CLSM Spectra Lab.

To further facilitate adaptation by the research community, we provide the analysis software *CLSMspectraLab* via https://github.com/ErikMaris/CLSMspectraLab, which interfaces with the freely available XANES Wizard software that is part of the *TXM-Wizard* software suite used to generate the material maps.[3]

The emission bands used in the main text correspond to 420–600 nm, 630–660 nm and 660–700 nm, which are bands 1, 2 and 3, respectively.

3 Supporting Figures and Table

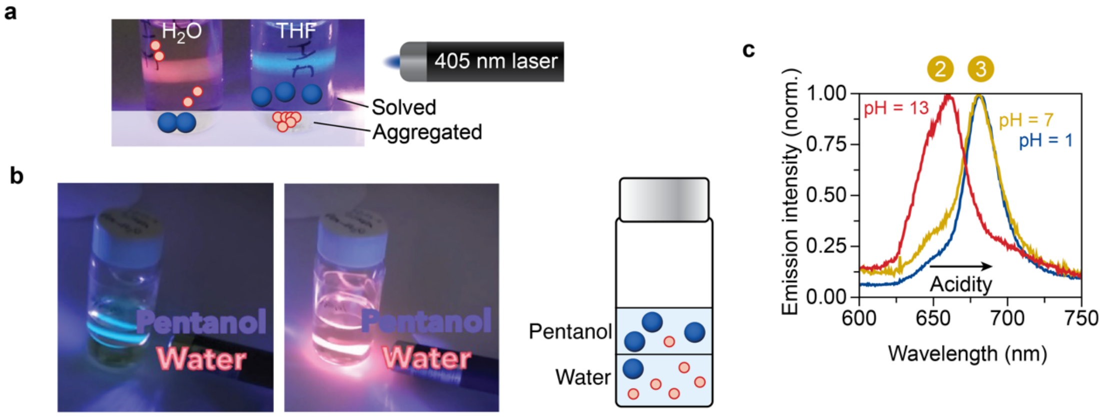

Figure S1 | Fluorescence emission properties of carbon dots and porphyrin in solution. a) Fluorescence emission of a carbon dot (CD)/porphyrin (PP) mixture (> 5 mg/mL) in water and THF, excited with a 405-nm laser. In water, the emission is red, indicating that the PP is water-soluble and hydrophilic, while the emission is blue in THF, demonstrating the amphiphilic nature of the CDs. We ascribe this observation to the quenching of the CDs and PP fluorescence when they are aggregated in less good solvents. Please note that the concentration of the CD/PP mixture is > 10× higher than during the staining experiments, favouring aggregation. b) CD/PP mixture in a water–pentanol, bi-phase solution under 405-nm excitation. By dissolving the CD/PP mixture in a two-layer system of immiscible liquids with different polarity, the partitioning of the CDs and PP in the two layers reveals their affinity for the hydrophobic phase (pentanol) or the hydrophilic phase (water). Pentanol solved blue emissive species, while water mainly solves the red emissive products. The partitioning is readily detected from the fluorescence-emission colour after excitation. We find that CDs have a strong affinity for hydrophobic and amphiphilic liquid phases (pentanol and THF, respectively) and PP for the hydrophilic phase (water). c) PL emission at 405 nm excitation shows the pH responsiveness of Bands 2 and 3 of porphyrin in aqueous solution.

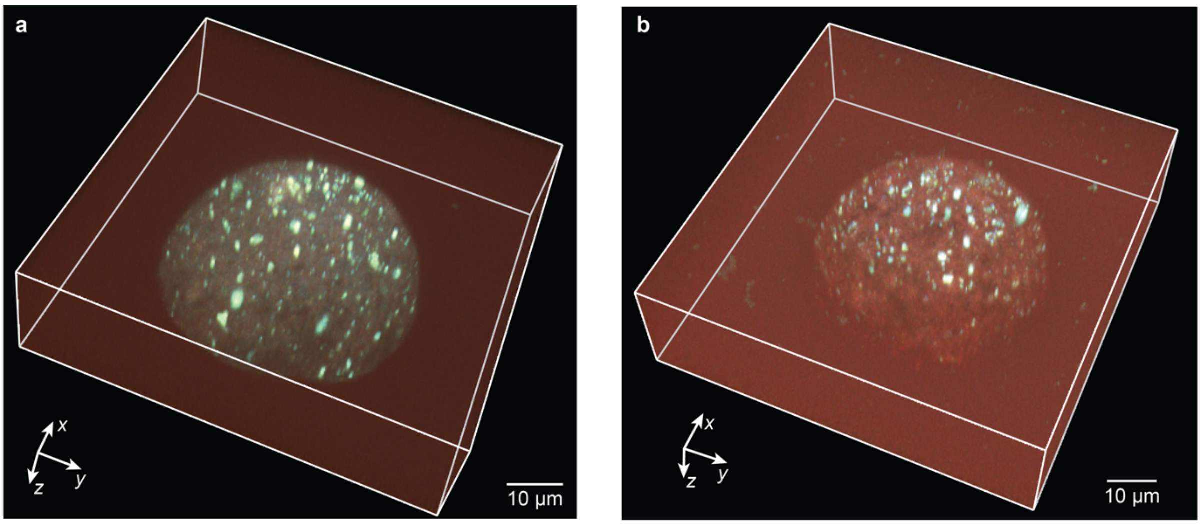

Figure S2 | Volume imaging showcase. Reconstructed volume from a stack of two-dimensional confocal laser-scanning microscopy image slices of two laboratory-made FCC particles after staining with the CD–PP mixture at pH 3 (a) and pH 7 (b). The scale bar represents the dimensions at the front of the box. The dimensions of the boxes are 86.8 × 86.8 × 22.0 µm^3 (a) and 88.9 × 88.9 × 25.0 µm^3 (b).

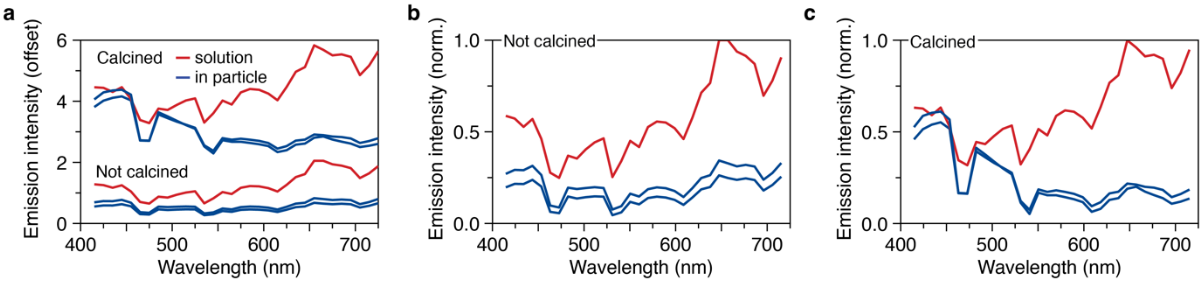

Figure S3 | Fluorescence spectra of stained silica particles. a–c) Fluorescence emission spectra recorded inside stained DML silica particles and in the surrounding solution. The stained silica particles were either untreated or calcined before staining. The carbon dots (Band 1: 420–600 nm) have a higher affinity for calcined silica. The responses of porphyrin (Bands 2 & 3: 630–700 nm) The spectra of the calcined samples were offset by 2 in (a) and normalized in (b,c).

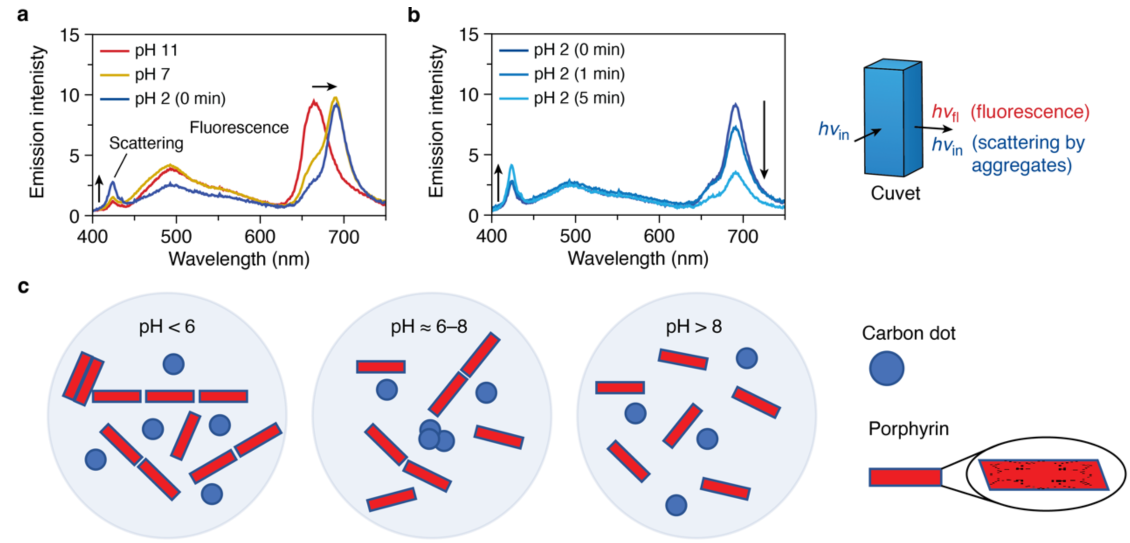

Figure S4 | J-aggregation of porphyrin in aqueous solution. a,b) Emission spectra of the carbon dot (CD) /porphyrin (PP) solution measured as a function of pH and over time. Increased scattering of the 405-nm laser source coincides with a red shift of the PP emission from pH = 11 to 7 to 2 in (a). Scattering increases over time at pH = 2 in (b), while the PP fluorescence signal decreases, indicating the growth of aggregates. This observation hints to an effect of aggregation on the PP emission at acidic pH in addition to the effect of protonation–deprotonation. The red-shift of the emission around 650–700 nm indicates that these aggregates are porphyrin J-aggregates. The intensity loss observed over time is typical for J-aggregation due to aggregation-induced quenching.[4] Note that the emission signal was obtained at an angle of 90° with respect to the incident light and therefore only fluorescence and scattering signal can reach to detector of the spectrophotometer. c) Schematic representation of carbon dot and porphyrin aggregation at different pHs. J-aggregation of porphyrin, and aggregation in general, is favoured in acidic environment, and can be recognised by the head-to-tail arrangement.

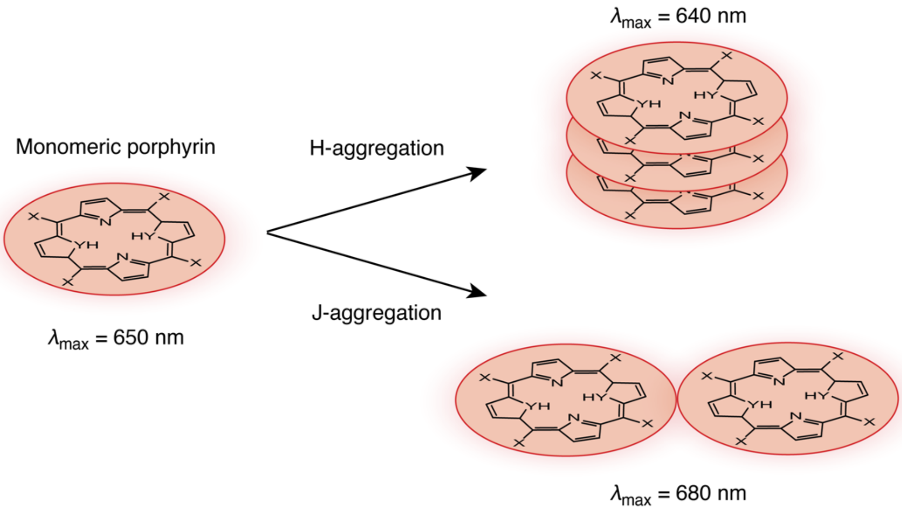

Figure S5 | Porphyrin J-aggregation emission characteristics. Schematic representation of H- and J-aggregation of the porphyrin derivative synthesized in this study, and its effect on the maximum fluorescence-emission wavelength. When the sample is highly aggregating in an acidic environment, an additional peak at 640 nm appears, which we ascribe to H-aggregation. The emission wavelength with the maximum intensity is denoted by λ_{max}.

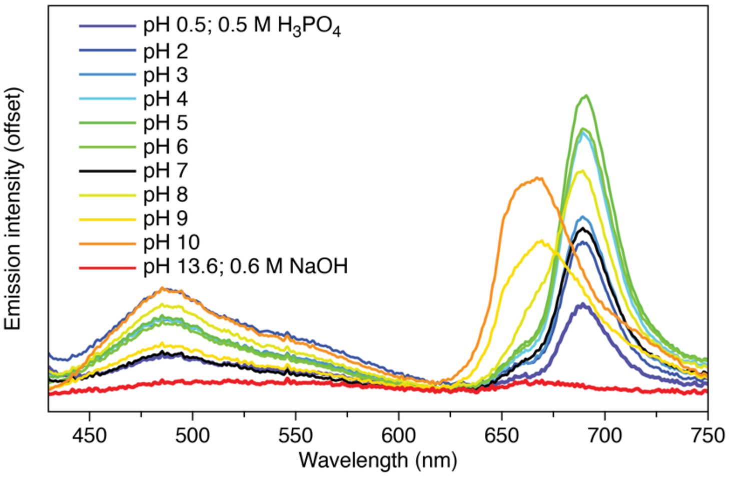

Figure S6 | Fluorescence-emission spectra as function of pH. Carbon dot (CD)/porphyrin (PP) mixture in aqueous solution measured as function of solution pH (λ_{ex} = 405 nm). pK_a of PP is around pH 8–9, where transition between band 2 (λ_{em} ≈ 650 nm) and 3 (λ_{em} ≈ 680 nm) occurs. At pH 13.6, the fluorescence is quenched, which does not follow the trend at milder alkaline conditions (i.e., pH = 8, 9, and 10).

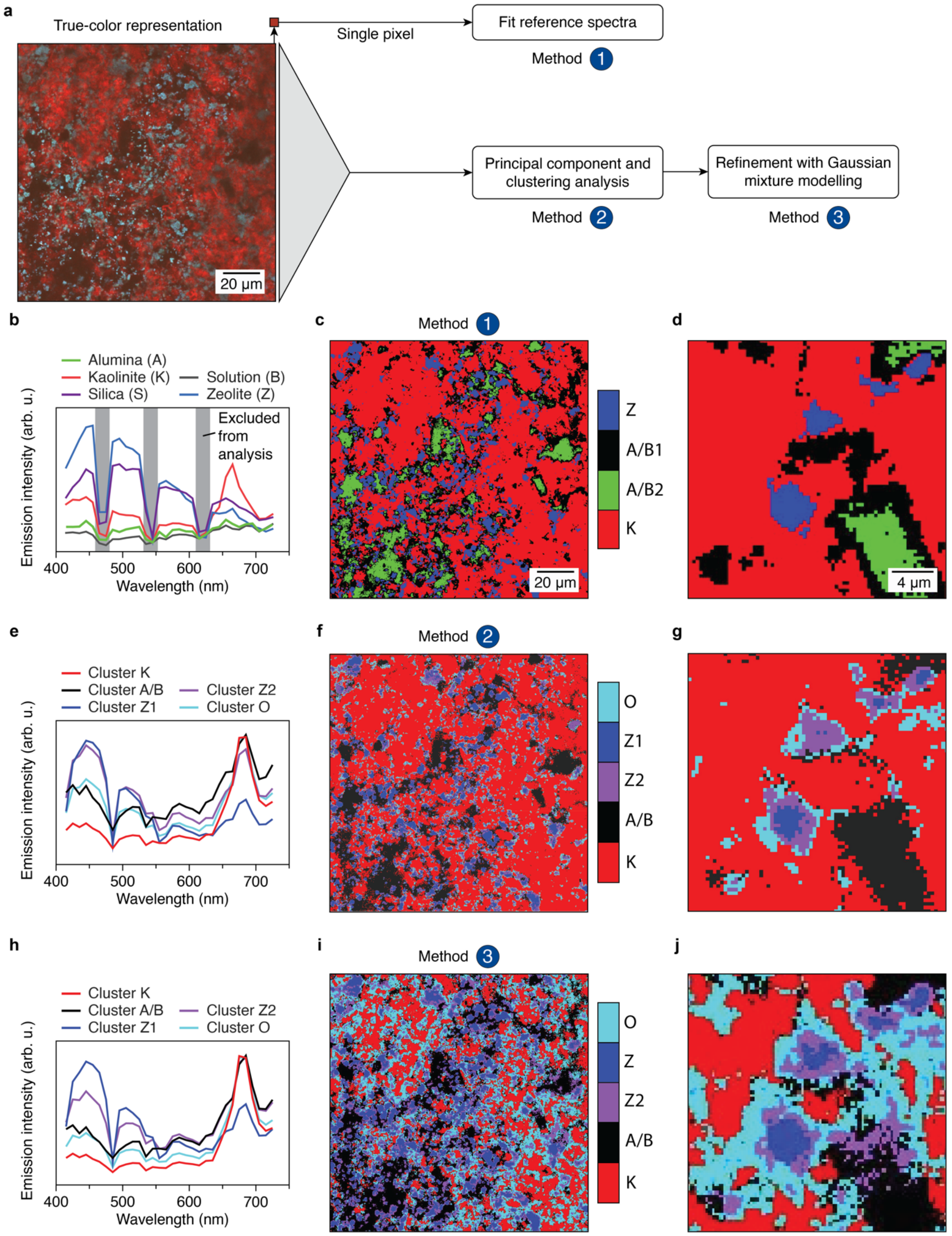

Figure S7 | Comparison of methods to generate material maps. a) True-colour confocal laser scanning microscopy (CLSM) image of an extrudate—containing USY, clay, and alumina binder—after staining by a carbon-dot/porphyrin solution at pH 7 (λ_{ex} = 405 nm). Each pixel in the CLSM image contains an emission spectrum in the range 415–735 nm, recorded with a step size of 10 nm. We evaluated three methods for the material assignment: comparison with a reference spectrum (Method 1), principal component analysis (PCA) followed by *k*-means clustering (Method 2), and PCA followed by clustering with a Gaussian mixture model (GMM,

Method 3). Method 1 is performed on a single-pixel basis, while Methods 2 and 3 are include all pixels in a global analysis. b–d) Method 1 fits reference spectra (b) to the single-pixel spectra. Data points excluded from the fit do not contain information as some wavelengths of the emitted fluorescence are blocked by the dichroic mirror. The resulting material map (c) and a magnification of a representative domain (d) are given. Overlap pixels are indicated with “O” (see Notes to **Figure S7** below). e–g) Using Method 2, principal component analysis and *k*-means clustering resulted in 8 clusters, which were merged in 5 clusters with qualitatively different spectra (e, no background correction). The resulting material map (f) and a magnification of a representative domain (g) are given. h–j) In Method 3, the clustering result of Method 2 was refined with Gaussian mixture modelling, which resulted in the 5 clusters with spectra given in (h, no background correction). The resulting material map (i) and a magnification of a representative domain (j) are given. The insets in (d,g,j) are of the same area.

Notes to Figure S7: A requirement for quantitative analysis is that all pixels in the CLSM image are assigned (or *classified*) to a host material. As each pixel contains a spectrum, the host material can be assigned on a per-pixel basis. **Figure S7a** summarizes the methods used to assess the content and distribution of an extrudate comprising kaolinite, alumina, and USY zeolite (composition details in **Table S1**, extrudate S1). Method 1 compares the single-pixel spectra with a database of reference spectra using least-squares fitting (**Figure S7b**). The pixel is assigned to the reference spectrum of the material with the smallest fit error, building up the material map given in **Figure S7b,c**. Because of the noise on the single-pixel spectra, this approach is more robust than a linear combination fit of all reference spectra. Method 2 relies on a reduction of the dimensionality of the single-pixel spectra with principal component analysis (PCA). The pixels are then clustered into groups with similar spectra using *k*-means clustering. The final clusters are then assigned manually to specific materials via their cluster spectrum (**Figure S7d**). The material map is given in **Figure S7e**. Finally, the Method 3 takes the clusters of Method 2 and refines the *k*-means result using expectation maximization (EM) for Gaussian mixture modelling (GMM). The refined spectra and material map are given in **Figure S7e,f**.

Altogether, here Method 2 is the preferred approach and results in high-quality material maps. The material maps generated with the three methods yield similar results but differ in the details. Our considerations to prefer Method 2 (in this case) are:

- PCA and clustering is robust to noise. This is important, because the single-pixel spectra are noisy, even after binning. A global-analysis method deals much better with noise than a per-pixel method, such as Method 1. Moreover, PCA reduces the dimensionality of the data before clustering, which reduces the noise in the data.
- PCA combined with *k*-means clustering detects pixels that contain more than one material (overlap pixels). This detection is not possible by using only reference spectrum (Method 1). We find that these interfacing pixels are prevalent and should not be ignored in the analysis.
- Refinement of the *k*-means clusters with GMM results in misassignment of the pixels and enlargement interfacing pixels domains (Method 2, **Figure S7f**). The pixel misassignment was checked by manual comparison with the unprocessed spectra.

Since the alumina phase is inaccessible (see **Figure S14**), it is not always possible to distinguish it from pores containing only solution, and assignment of either phase to a spectrum is ambiguous.

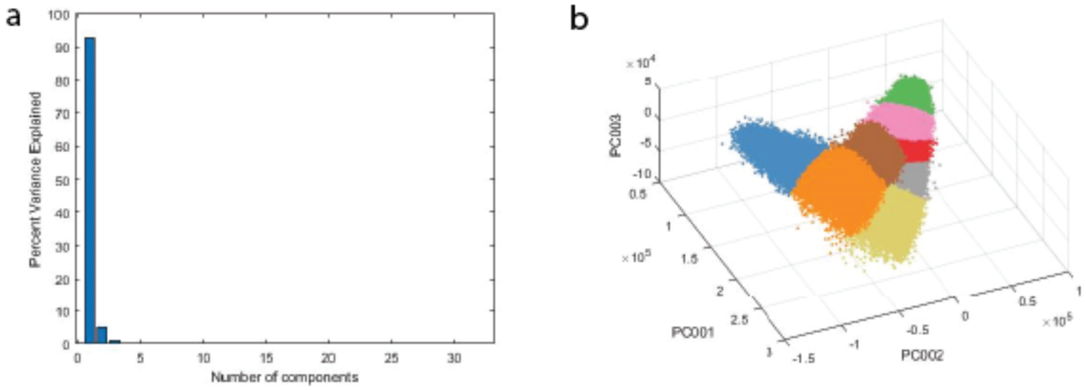

Figure S8 | Principal component analysis and clustering. a) Scree plot of the principal component analysis (PCA) showing the percentage of variance explained by the principal components (PCs). Most of the information (variance) in the dataset is in the first three PCs; therefore, clustering was performed using these three PCs. b) Plot of eight clusters in PC space before merging them into five larger ones based upon inspection of the resulting cluster spectra.

Notes to Figure S7: in XANES Wizard, principal component analysis was used to reduce the dataset to three principal components using the scree plot and the “elbow” criterion (**Figure S7a**). Then, *k*-means clustering was performed by choosing eight clusters (intentional over-clustering) and two replicates (**Figure S7b**). After clustering, the eight clusters were merged to five ones by inspecting the 3D plot of clusters in principal component space and the spectra of each cluster. Finally, each cluster was assigned to a specific phase by comparing the spectra of the clusters with reference spectra of each phase.

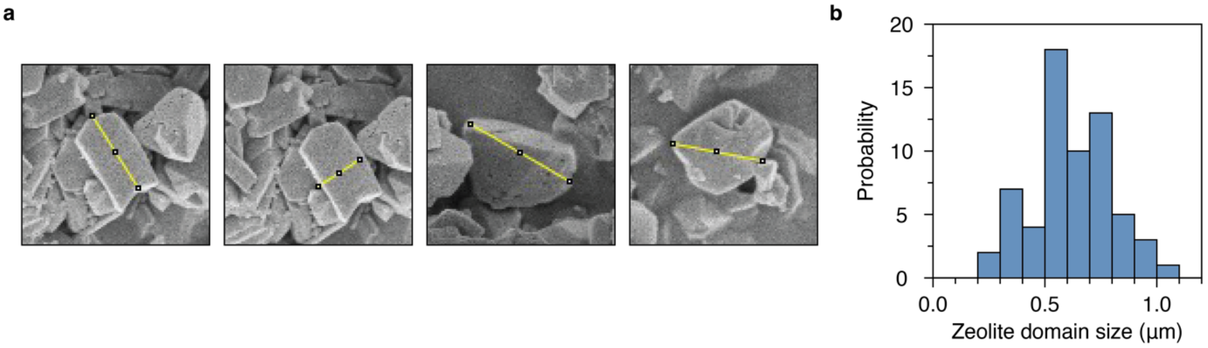

Figure S9 | Size determination of individual crystallites with scanning electron microscopy (SEM). a) Examples of size measurements of individual zeolite crystallites from SEM images. Only isolated or well recognizable crystals (with sharp edges) are selected for particle size analysis. The longest diameter and the diameter perpendicular to this were measured—when possible—see first two panels. b) Histogram of the measured zeolite crystallites using the method in (a) based on 63 data points.

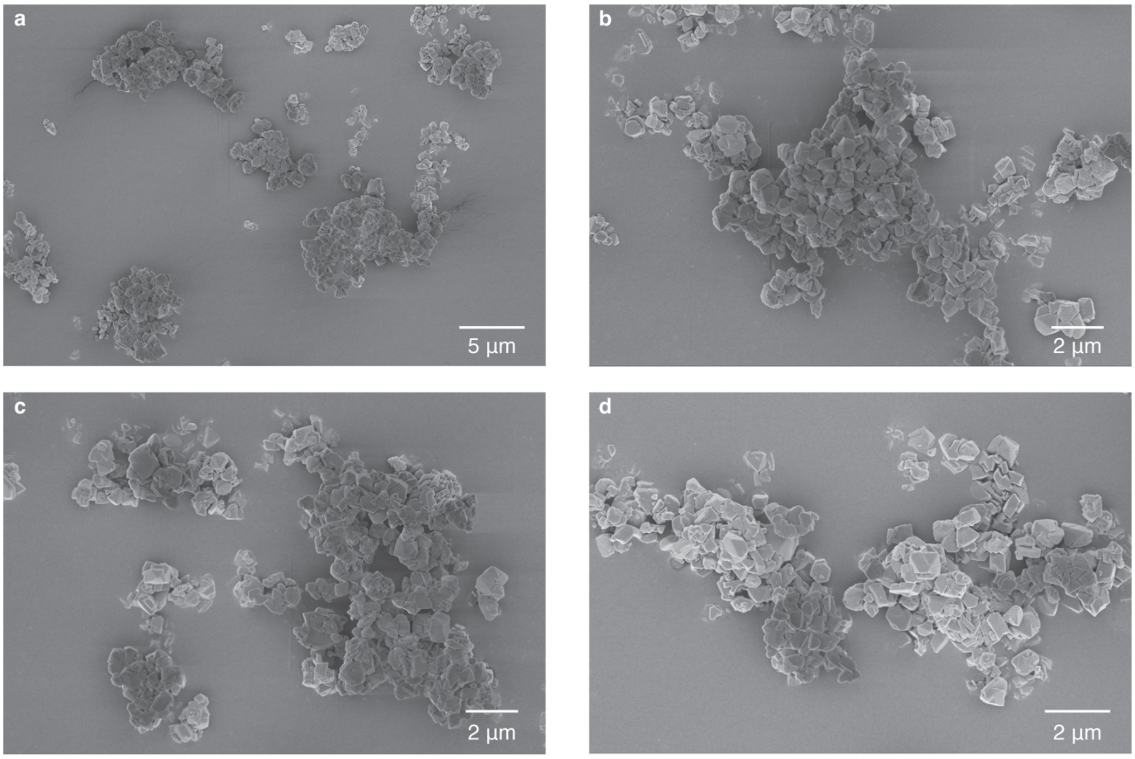

Figure S10 | Scanning electron microscopy (SEM) images of zeolite. a–d) SEM images of the pristine ultrastable Y zeolite, which was used to make the catalyst extrudates and spray-dried catalyst particles. The zeolite same consists of small, submicron zeolite crystallites, which are intergrown forming micron-sized agglomerates.

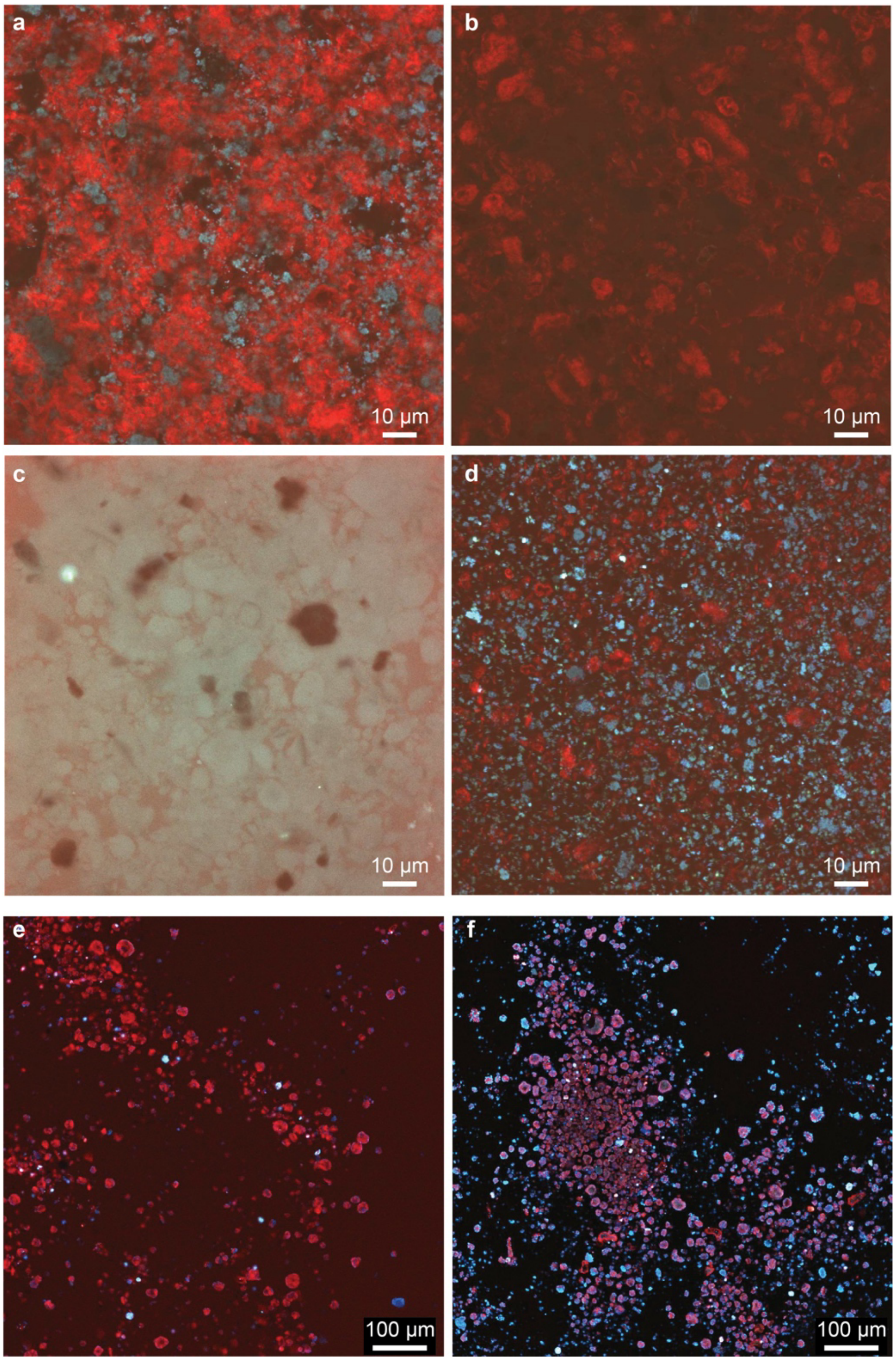

Figure S11 | Real-colour images of catalyst extrudates and spray-dried catalyst particles. Confocal laser scanning microscopy (CLSM) true-colour images of catalyst extrudates (Extrudates 1–4, composition in **Table S1**) and spray dried catalyst particles (from Slurries 1–2, composition in **Table S3**): a) Extrudate S1; b) Extrudate S2; c) Extrudate S3; d) Extrudate S4; e) Slurry 1; and f) Slurry 2.

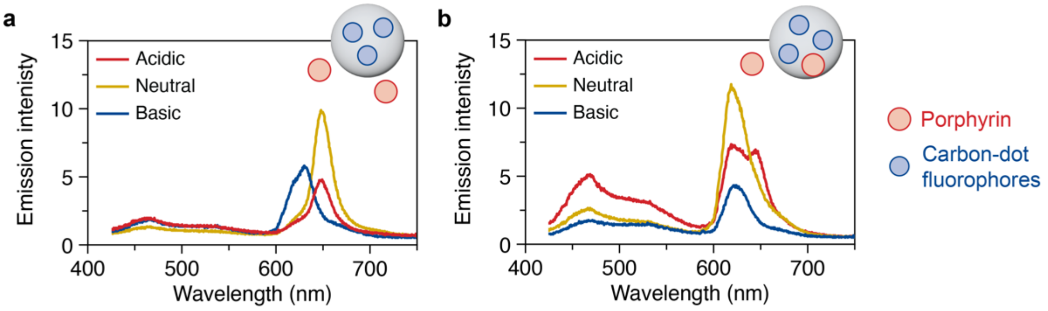

Figure S12 | Loss of porphyrin pH responsiveness at longer synthesis times. a,b) Fluorescence emission spectrum of the porphyrin (PP)/carbon dot (CD) system for 3 h (a) and 18 h (b) solvothermal treatment. The acidic conditions were 0.5 M H_3PO_4 and the basic conditions 0.5 M NaOH. A loss of the pH-responsiveness of the PP constituents is observed. A schematic of the proposed mechanism is shown on top: the PP loses pH-responsiveness because of its (partial) encapsulation in the amorphous CD, shielding it from the solution.

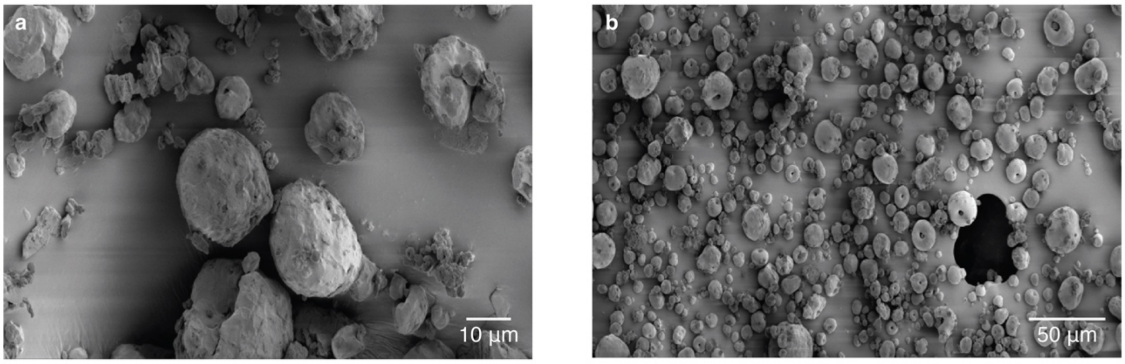

Figure S13 | Scanning electron microscopy (SEM) images of spray dried particles. a–b) SEM images of spray-dried particles synthesised with Slurry 1 (a) and Slurry 2 (b).

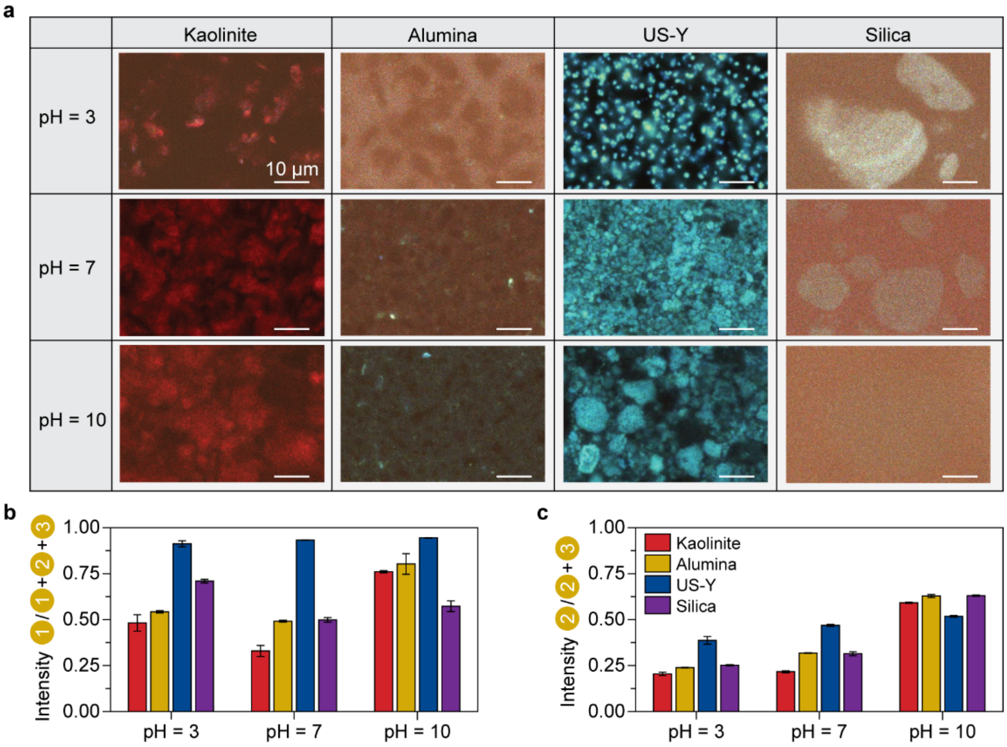

Figure S14 | Selecting the staining solution pH to maximize contrast and resolvability. a) Representative confocal laser scanning microscopy (CLSM) images of silica, ultrastable-Y (USY) zeolite, kaolinite, and alumina reference materials 25-mM-PBS buffered at pH 3, 7, and 10. The fluorescence emission spectrum is plotted as a real-colour image and the scale bars are 10 µm. b) Intensity ratio of Band 1 (420–600 nm) and Bands 1 + 2 + 3 (420–700 nm) of data in a). c) Intensity ratio of Band 2 (620–660 nm) and Bands 2 + 3 (620–700 nm) of data in (a). The error bars in (b,c) are the per-pixel standard error over three frames recorded at different positions in the sample. At pH = 7, we find the optimum trade-off between high signal and sufficient spectral differences between the different materials. Please note that the spectral resolvability of the alumina and silica is poor at this pH, but we can identify alumina via its absence of porosity.

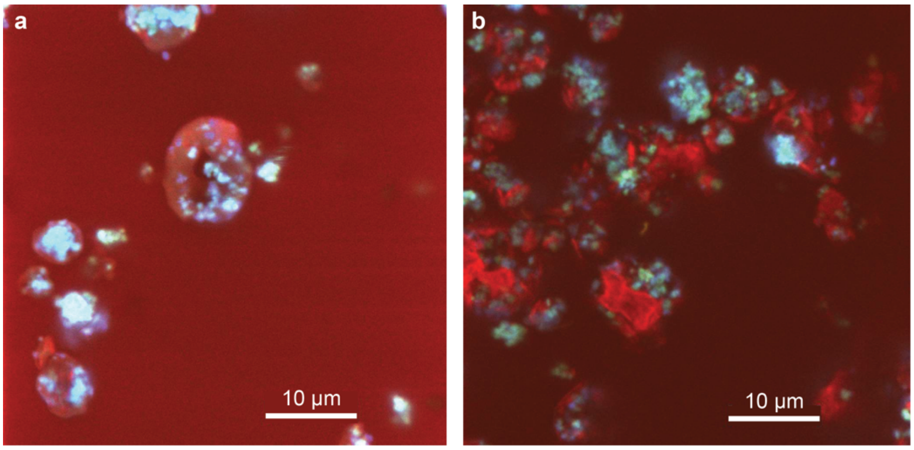

Figure S15 | pH buffering improves resolvability in confocal fluorescence microscopy images. a–b) Confocal laser scanning microscopy images of carbon dot (CD)/porphyrin (PP)-stained spray-dried catalyst particles containing silica, ultrastable Y (USY) zeolites, alumina, and kaolinite (see also Figure 2b, left). The staining solution was either in non-buffered, Milli-Q water (a) or in 25 mM PBS buffered at pH = 7 (b). pH buffering of the solution resulted in improved resolvability of features in the images. This can be understood as following: a solid in aqueous solution tends to change the pH of the solution towards its isoelectric point (IEP). To reduce the distance from the solid surface at which the solution pH is affected, the solution is buffered. Moreover, the addition of the buffer increases the ionic strength of the system, which in turn reduces the range of the electrostatic interactions. This can increase the accessibility of the host by reducing repulsive and/or attractive interactions,[5] and hence, promote CD and PP uptake.

Table S4 | Isoelectric points of different oxide materials. The isoelectric point (IEP) in literature as well as the pH of 100 mg of solid dispersed in 1 mL water. For the measured values, the standard deviation of three measurements is given. Please note that there is a difference between IEP, which relates to the net charge measured by electrokinetic methods (e.g., Zetasizer), and the point of zero charge (PZC), which is related to the surface charge density and is usually measured by titration. However, they are similar for the investigated oxides. As more measurement data is available for IEP, specially for the clay and zeolite, we therefore reported the IEP.

Compound	Nominal IEP (range of IEP and PZC)	References	Measured pH of aqueous dispersion (100 mg in 1 mL)
Al_2O_3	9 (8.5–9.6)	6,7	6.12 ± 0.01
Cr_2O_3	7 (6.1–8.3)	6,7	5–8 (Ref. 8)
Kaolinite	3 (3–6.5)	9 (silica facet)	5.41 ± 0.17
MgO	9.8 (9.8–12)	6	8.11 ± 0.17
SiO_2	4 (< 3–5)	6	6.46 ± 0.07
TiO_2	6 (5.2–6.8)	6	6.05 ± 0.04
V_2O_5	1.5 (1–3)	6	4.0 (Ref. 10)
CBV780	2.55	11	2.70 ± 0.04
ZnO	9.2 (8–9.8)	6	5.43 ± 0.21

Table S5 | Advantages, limitations, and scope. Summary of the advantages, limitations, and scope of the carbon dot (CD)–porphyrin (PP) nanoprobes imaged with confocal laser scanning microscopy (CLSM).

Advantages	Limitations
**Complementary contrast.** The nanoprobes are sensitive to the surface properties and pore size of the host, which often does not provide contrast in electron and X-ray microscopy. This enables the rapid identification of, e.g., silica, kaolinite and zeolite, which are hard to distinguish with energy-dispersive X-ray spectroscopy (EDS) or Z-contrast.	**Mediocre resolution.** The resolution is diffraction limited (200 nm in the lateral direction at best), while electron and X-ray imaging provide at least an order of magnitude higher resolution.
**Surface sensitive.** The technique selectively images the accessible surface and pore space, which is usually the most interesting domain for catalysis and sorption applications.	**Limited imaging depth.** Measurements deep inside a sample are not yet recommended, because light scattering distorts the emission spectra and reduces signal intensity. This limitation can be overcome by using the multiprobe system in a refractive-index-matched solvent.
**Imaging in 2D and 3D.** CLSM enables imaging at a single focal plane inside the sample. By stacking a set of 2D scans, a reconstruction of the sample volume can be made.	
**Short imaging times.** Hyperspectral imaging with CLSM is relatively fast (~15 minutes per hyperspectral image) with respect to hyperspectral techniques like (SEM based) EDS (order of hours per elemental map).	
**Recommended applications.** The method is suitable for heterogeneous materials, where the functional domains have a different surface property, such as wettability, and/or pore accessibility. Another application is the study of homogenous materials, where the surface properties and/or pore accessibility changes for other reasons, such as a post synthesis treatment. We have identified heterogeneous porous materials used in the fields of catalysis and sorption as primary application. Particularly experiments demanding a high throughput and/or when no access to expensive electron and X-ray microscopy facilities is available.	**Application limitations.** The method is not suitable for applications where a <200 nm resolution is required, the volume rather than the surface should be imaged, for samples that are unstable in water, for non-porous samples, and/or for samples without variations in the surface properties that enable the adsorption of CDs and/or PP, such as some metal oxides (see Fig. 2a).

4 References

1. Ganjkhanlou, Y. *et al.* Dual Fluorescence in Glutathione-Derived Carbon Dots Revisited. *J. Phys. Chem. C* **126**, 2720–2727 (2022).
2. Schindelin, J. *et al.* Fiji: an open-source platform for biological-image analysis. *Nat. Methods* **9**, 676–682 (2012).
3. Liu, Y. *et al. TXM-Wizard*: a program for advanced data collection and evaluation in full-field transmission X-ray microscopy. *J. Synchrotron Radiat.* **19**, 281–287 (2012).
4. Maris, J. J. E. *et al.* Molecular Accessibility and Diffusion of Resorufin in Zeolite Crystals. *Chem. Eur. J.* **30**, e202302553.
5. Wu, H., Sarfati, R., Wang, D. & Schwartz, D. K. Electrostatic barriers to nanoparticle accessibility of a porous matrix. *J. Am. Chem. Soc.* **142**, 4696–4704 (2020).
6. Kosmulski, M. Isoelectric points and points of zero charge of metal (hydr) oxides: 50 years after Parks' review. *Adv. Colloid Interface Sci.* **238**, 1–61 (2016).
7. Nobbmann, U. Isoelectric points of Nanomaterials – Q&A. *Malvern Panalytical* https://www.malvernpanalytical.com/en/learn/knowledge-center/insights/isoelectric-points-of-nanomaterials-qa (2017, accessed on 11-09-2024).
8. Chromium III oxide | 1308-38-9. *Chemical Book* https://www.chemicalbook.com/ChemicalProductProperty_EN_CB8394705.htm (accessed on 11-09-2024).
9. Kumar, N., Andersson, M., van den Ende, D., Mugele, F. & Sîretanu, I. Probing the surface charge on the basal planes of kaolinite particles with high-resolution atomic force microscopy. *Langmuir* **33**, 14226–14237 (2017).
10. Armaković, S. J. *et al.* Photocatalytic Activity of the V_2O_5 Catalyst toward Selected Pharmaceuticals and Their Mixture: Influence of the Molecular Structure on the Efficiency of the Process. *Molecules* **28**, 655 (2023).
11. Tran, H. N., Viet, P. V. & Chao, H.-P. Surfactant modified zeolite as amphiphilic and dual-electronic adsorbent for removal of cationic and oxyanionic metal ions and organic compounds. *Ecotoxicol. Environ. Saf.* **147**, 55–63 (2018).
